# Climate Change Impacts on Environmental and Human Exposure to Mercury in the Arctic

**DOI:** 10.3390/ijerph120403579

**Published:** 2015-03-31

**Authors:** Kyrre Sundseth, Jozef M. Pacyna, Anna Banel, Elisabeth G. Pacyna, Arja Rautio

**Affiliations:** 1 NILU-Norwegian Institute for Air Research, Department of Environmental Impacts and Economics, Instituttveien 18, P.O. Box 100, NO-2027 Kjeller, Norway; E-Mails: jp@nilu.no (J.M.P.); anb@nilu.no (A.B.); ep@nilu.no (E.G.P.); 2 Department of Analytical Chemistry, Gdansk University of Technology, Chemical Faculty, PL 80-952, Gdansk, Poland; 3 Center for Arctic Medicine, Thule Institute, P.O. Box 7300, University of Oulu, FI-90014 Oulu, Finland; E-Mail: arja.rautio@oulu.fi

**Keywords:** mercury, climate change, Arctic, Europe, policymaking, exposure, health

## Abstract

This paper reviews information from the literature and the EU ArcRisk project to assess whether climate change results in an increase or decrease in exposure to mercury (Hg) in the Arctic, and if this in turn will impact the risks related to its harmful effects. It presents the state-of-the art of knowledge on atmospheric mercury emissions from anthropogenic sources worldwide, the long-range transport to the Arctic, and it discusses the likely environmental fate and exposure effects on population groups in the Arctic under climate change conditions. The paper also includes information about the likely synergy effects (co-benefits) current and new climate change polices and mitigation options might have on mercury emissions reductions in the future. The review concludes that reductions of mercury emission from anthropogenic sources worldwide would need to be introduced as soon as possible in order to assure lowering the adverse impact of climate change on human health. Scientific information currently available, however, is not in the position to clearly answer whether climate change will increase or decrease the risk of exposure to mercury in the Arctic. New research should therefore be undertaken to model the relationships between climate change and mercury exposure.

## 1. Introduction

Mercury (Hg) is a toxic, persistent and mobile pollutant. In methylated form (methylmercury‒MeHg), it bio-accumulates and bio-magnifies in the environment and ultimately becomes a threat to human health via the food chain. Methylmercury passes through the placenta and the blood-brain barrier, so methylmercury exposure to women of childbearing age and to their children is of major concern. Moreover, mercury levels continue to rise in some regions of the world. As a result, several studies have been conducted on the behaviour of mercury in the environment and its environmental and economic consequences [1,2]. The potential for climate change to affect the environmental transport of mercury and its risks of human health impacts, has recently gained increased attention from the international scientific community. Although, climate change effects on mercury have previously been studied [3,4,5], the recent signing and implementation of the Minamata Convention has pushed for a re-examination of the topic, especially with focus on the mercury contaminant sources. In this connection, the knowledge on how mercury contamination can be limited and how to assess climate effects, allows for identification of suitable strategies that can be prepared for adaptation and for the prevention of adverse health outcomes related to climate-mediated changes [6].

The Arctic is particularly sensitive to climate change, compared to other regions. Investigating climate change effects on mercury contamination in the Arctic may therefore present an opportunity to gain insight into changes that may later impact populations in other regions of the world, such as in Europe. This was the basis for the European Commission funded Arctic Health Risks project (ArcRisk; www.arcrisk.eu) where the main goal was to investigate if climate change in the form of increased temperature would affect the transport of contaminants to the Arctic. ArcRisk research activities looked at the linkages between environmental contaminants, climate change and human health with the aim to assess strategies for adaptation and prevention of adverse health outcomes related to climate-mediated changes in exposure to pollutants in human populations in the Arctic and in Europe. Describing the full sequence from emissions, all the way through to human exposure and health impacts of contaminants with varying emission sources, chemical properties and effects, is complex. The Arcrisk project recognized that the scientific tools and methods currently available, would not allow detailed quantification of all the involved steps in this sequence, or the potential influence of all aspects of climate change on these steps. Scientific information was judged, however, to be available for mercury and polychlorinated biphenyls (PCBs) since research on emissions, environmental transport and transformations, as well as exposure and health effects of these compounds has been ongoing for decades and our knowledge of these is considerably greater than for other contaminants. A review of mercury and polychlorinated biphenyls was therefore chosen as case studies to illustrate how climate change can be expected to influence pollutant behaviour, fate and human health impacts [7].

This paper presents the outcome of the ArcRisk mercury review. It presents the state-of-the art of knowledge on atmospheric mercury emissions from anthropogenic sources worldwide, the long-range transport to the Arctic, and it discusses the likely environmental fate and exposure effects on population groups in the Arctic under climate change. It also presents likely synergy effects (co-benefits) current and new climate change polices and mitigation options might have on mercury emissions reductions in the future. The information presented in our mercury review builds upon data collected and assessed by the project partners in the EU ArcRisk project, results published from the scientific literature as well as recent works compiled in international assessment reports, e.g., performed in the Arctic Monitoring and Assessment Programme (AMAP) and United Nations Environmental Programme (UNEP) assessments.

## 2. Current Status of Environmental Mercury Contamination

### 2.1. Major Drivers of Mercury Contamination

Mercury found in the Arctic is a result of worldwide human activities and natural processes. Production and consumption of energy and industrial goods, increasing human population and their related activities and demands are the main drivers of environmental change, and affect mercury concentrations in the Arctic and Europe at present and will continue to do so in the future. Emission inventory results presented in the 2013 global mercury assessment prepared by UNEP and AMAP have identified artisanal and small- scale gold mining as the major anthropogenic sources of global mercury emissions to the atmosphere in 2010, followed by combustion of fossil fuels (mainly coal) for energy and heat production in power plants and for industrial and residential purposes [8]. These sources account for more than half of the anthropogenic mercury emissions globally, estimated to about 1960 tonnes annually, within the uncertainty range of 1010–4070 tonnes. Other significant industrial sources of global mercury emissions to the atmosphere are primary non-ferrous metals production, cement production, large-scale gold production and waste from consumer products (mostly landfill but also incineration), pig iron production, chlor-alkali industry, mercury production, oil refining and dental amalgam through human cremation [8].

Mercury used for artisanal gold mining, and mercury present as impurity in various fuels and ores, as well as in limestone and other raw materials in cement manufacture, are the key parameters affecting the amount of atmospheric emissions of mercury from the above mentioned sources [8]. Details on the impact of various factors on the magnitude of emissions are presented in the literature [9]. Although the total anthropogenic mercury emissions to the atmosphere appeared to be relatively stable between 1990 and 2010 [8,10], a decrease in emissions in Europe and North America during the time period has been offset by an increase in Asia. In general, the largest increase in emissions is due to an increase in coal combustion for power and heat generation and for industrial purposes. Increased use of air pollution controls, removing mercury as a co-benefit in combination with some mercury specific removing technologies, has slowed down or even reduced the emissions from the increased energy demand. This is the case for Europe and North America, but is also becoming apparent from new coal-fired power plants with state-of-art pollution controls recently installed in China [8].

Mercury emitted naturally from volcano eruptions, geothermal sources, topsoil and re-emission of the contaminant from aquatic and terrestrial surfaces adds large amounts to the global mercury balance. Re-emitted mercury originates from both natural and anthropogenic sources cycling between air, land, and water; however, anthropogenic sources are the main contributor to the environmental burden of mercury, causing higher levels of re-emissions. Over the years, several estimates have been presented in the scientific literature on the annual magnitude of such natural and re-emitted anthropogenic emissions, ranging from 3600 tonnes per year [11] to 5300 tonnes [12]. Other studies include estimates of 4200 tonnes [13], 4278 tonnes [14], 4532 tonnes [15], 4800 tonnes [16], and 5207 tonnes [17]. A Monte Carlo analysis recently performed in the EU GMOS project (www.gmos.eu) of the upper and lower bounds of mercury emissions as reported in several published papers and international reports (*i.e.*, [15,17,18]) supports an even higher estimate of about 6200 tonnes [19].

### 2.2. Major Pathways of Mercury to the Arctic with Air Masses and Water Currents

The amount of the mercury transported to the Arctic depends on the emissions of total mercury and their chemical forms, emission dispersion conditions, and the meteorological parameters. Most mercury reaches the Arctic from distant sources via the atmosphere and via rivers and ocean currents, with the atmosphere and upper oceans being the dominant pathways [5]. Transport simulations (e.g., GRAHM, Environment Canada; GLEMOS, Meteorological Synthesizing Centre-East; DEHM, Danish National Environmental Research Institute; DMU/NERI and GEOS-Chem) have identified Arctic contamination to originate from outside the region, mainly from sources in the Northern Hemisphere (Europe and North America) and from East- and South Asia. North American and East Asian impact is slightly greater in the western Arctic while European sources, being closer to the Arctic, influence greater over the European Arctic. South Asian impact is seen over Greenland and adjacent oceans resulting from dominant air transport pathways [5,20].

About 100 tonnes of mercury are delivered to the Arctic Ocean from the air each year, which is about the same inflowing amount from the Atlantic and Pacific Oceans, rivers and coastal erosion [21,22,23,24,25,26]. These inputs already comprise inorganic and small amounts of methylated and dissolved gaseous mercury resulting from transformations that occurred in these reservoirs even before the mercury reached the Arctic environment. During spring flood, boreal rivers transport large quantities of mercury from the continents (e.g., Siberia) to the Arctic Ocean. The Arctic Ocean seawater accumulates about 25 tonnes of mercury each year [5]. The budget calculations suggest that about 75 to 90 tonnes of mercury are exported from the Arctic Ocean in ocean outflow each year and that about 110 tonnes are deposited in Arctic Ocean shelf and in deep ocean sediments [5,25]. Trough measurements and flux estimates, an input of 80 tonnes per year of river total mercury to the portion of Arctic marine waters has been found between 70°–90° N [27,28]. Fisher *et al.* [27] further report that atmospheric net deposition (45 tonnes) and coastal erosion (15 tonnes per year) follows river inputs as the most important sources of mercury. Evasion of mercury from the surface ocean (90 tonnes per year) and particle settling (43 tonnes per year) they claim to be the most important processes removing mercury from surface ocean waters. Evasion of mercury from the surface ocean has furthermore been proposed to explain the summertime peak in concentrations observed in the Arctic atmosphere [27,29].

### 2.3. Concentration Levels of Mercury in Various Components of the Arctic Environment

Arctic abiotic environments generally exhibit lower levels of contamination than those found in regions closer to major emission sources, such as in most of Europe. However, certain Arctic attributes, such as cold, ice and snow cover, extended periods of darkness, mean that the Arctic has the potential to accumulate certain globally transported mercury. Concentration profiles in peat, sediments and ice cores in the Arctic show that there is an increased deposition of mercury today compared to the pre-industrial period, with a peak deposition occurring between the 1950s and the 1970s [30]. Recent (ten-year) trend analysis of atmospheric mercury, has shown that mercury in ambient air behaves differently on both long and short term. For high Arctic mercury observation sites such as the Alert (Nunavut, NU, Canada) and the Zeppelin station (Svalbard, Norway), a different temporal pattern has been observed for mercury in the ambient air compared to sub-Artic stations (Kuujjuarapik in Nunavik) and mid-latitude sites (St. Anicet in Quebec, Kejimkujik in Nova Scotia, and Egbert in Ontario). At Alert, a small gaseous elemental mercury (GEM) decreasing trend (0.9% reduction per year) has been observed whereas at the Zeppelin station, no trend has been observed. Faster decreases were observed at the remaining sites [20]. Previous discussions have indicated that changes in mercury concentrations observed in air and wet deposition at long-term monitoring stations are reflecting changes in re-emission of mercury from surface ocean and soil reservoirs [31,32].

Large quantities of mercury accumulated during previous millennia, including recent emissions from human activities, are stored in Arctic permafrost, soils, sediments and glaciers. Mercury in surface snow is mainly found in its oxidized form (Reactive Gaseous Mercury—RGM), with concentrations ranging from a few up to hundreds of ng/L. The rate of deposition of mercury onto the snow is greater in Arctic coastal areas than in more southerly locations and inland areas [33,34]. Studies of total gaseous mercury (TGM) concentrations in snowmelt water in the Canadian and Greenland Arctic generally display a range from 0.3 to 10 ng/L, with an average of about 3 ng/L [25], although high mercury concentrations may occur briefly in the surface melt-water from a snowpack during the earliest melt period (e.g., up to 24 ng/L for 1 day; [35]). Table 1 presents the mercury concentrations in selected compartments of the Arctic environment.

**Table 1:** Mercury concentrations in selected compartments of the Arctic environment.

Media	Concentration	Unit	Reference
Ambient air *	≈1.5	ng/m^3^	[20]
Ocean	0.1–3	pg/L	[36]
Ice free surface seawater	3–52	pg/L (DMHg)	
	15–68	pg/L (MMHg)	
	15–49	pg/L (GEM)	
Snow			
Air-25 km flight-rime	15,500	ng/L	
Air- rime (2005 year)			
Air-rime (2006 year)	5210	ng/L	
Air-condensate	1580	ng/L	
Sea-ice surface hoar	240	ng/L	
Sea-ice frost flowers	251	ng/L	
Terrestrial ground diamond dust	140	ng/L	
	476	ng/L	

* Alert and Zeppelin stations (monthly median TGM).

AMAP concludes that some Arctic animal species, in particular marine top predators, such as toothed whale, polar bears and some bird species, experience levels of mercury in their tissues and organs that are believed to exceed thresholds for biological effects [5]. The Arctic marine food webs consist of seven functional groups spanning five trophic levels that include primary producers (such as ice algae or phytoplankton), grazers, predatory invertebrates, fish, predators (such as beluga whales and seals), and apex predators (such as polar bears and humans) [28]. In ice algae and phytoplankton, mercury concentrations can range from a few up to hundreds of ng/g dry weight (dw), although lower concentrations have been observed in the winter season [28]. Many fish species and marine birds that feed in surface or subsurface waters display varying levels of methylmercury [37,38,39,40,41,42]. Estuarine and coastal fish may also display heightened concentration of methylmercury [43,44]. Mercury concentrations of Arctic cod range from 0.05 µg/g·dw [45] to 0.37 µg/g·dw, although higher concentrations have been observed in spring under the ice in the Amundsen Gulf/Franklin Bay [46]. Beaufort Sea benthic species (that comprise an important part of the beluga diet) have displayed mercury levels ranging from 0.2 µg/g·dw in flounders and predator invertebrates (such as shrimps), to 0.5 µg/g·dw in sculpins [28,46]. The highest mercury concentrations in polar bear hair have been found in the northern Canadian Arctic (such as in Lancaster Sound (3.1–73 µg/g) and in the Hudson Bay (2.6–8.7 µg/g)), compared to somewhat lower levels observed in Svalbard (0.2–3.9 µg/g) [47,48,49,50]. Blood levels of total mercury (THg) in polar bears from Alaska ranges from 7 to 213 μg/L [50]. In harp seal from Labrador and St. Lawrence, mercury concentrations have been found to increase with age (10 and 150 µg/L for male pups and adults, respectively). Some bird species such as ivory gull and black guillemot from Canada have mercury levels in their eggs sufficiently high (1.61 and 0.75 µg/g, respectively) to raise concern about negative effects on reproductive ability [5,51]. A recent study on blacklegged kittiwakes from Svalbard has suggested that high mercury concentrations could affect the ability of long- lived birds to skip breeding and thus affect their population dynamics [52]. Arctic fish muscle (especially marine species) generally displays mercury concentrations below suggested toxicity thresholds, and the guideline limits are only exceeded in a few species (e.g., 1.3 µg/g for landlocked Arctic char fish in Canada) [5,53].

### 2.4. Impacts of Mercury Pollution on Human Health in the Arctic

Maintaining traditional lifestyles of Northern Peoples with respect to diet composition exposes the indigenous populations to the high concentrations of mercury, giving rise to health concerns [8]. Chronic exposure to methylmercury via consumption of fish and sea mammals is a major concern for human health, especially developmental exposure that may lead to neurological alterations. This type of toxicity is characterized mainly by ataxia, dysarthria, paraesthesia, constraints in the visual field, hearing loss and disturbances in the development of nervous system [54,55,56,57,58]. Castoldi *et al.* [59] summarized the results of large-scale epidemiological studies concerning child development (at age of 2 weeks, 12 months, 7 and 14 years) and neurological disabilities in relation to in utero exposure to methylated mercury in various fish eating communities worldwide. Neurological disabilities including language, learning, and attention deficits and, to a lesser extent, motor and visual-spatial impairment have been reported due to the exposure to methylmercury. High dietary methylmercury exposure is suspected to reduce birth weight [60]. Methylmercury enhance formation of hydrogen peroxide and consequently the production of lipid peroxides and reactive hydroxyl radicals, which may alter membrane structure or disrupt mitochondrial function [60]. There is also evidence of the elevated risk for cardiovascular diseases and events, especially myocardial infarction. In the case of severe exposure, there is a risk of reproductive outcomes, immune system effects and premature death [61,62].

Based on the concentrations measured in human blood from Disko Bay (Greenland), Nuuk (Greenland), and Nunavik (Quebec, Canada) during the years 1992–2007, populations exhibited total mercury levels above the safety limit (blood) of 5.8 µg/L. The dietary surveys (from 1999 to 2002) in north-western Alaska have shown that the total mercury levels of fish consumers were considerably lower, but still higher (4.2 μg/L in blood) when compared to those of non-fish consumers (less than 1 μg/L) [63]. In the human populations of the Canadian Arctic, mercury levels in humans have declined by up to 50% over the past eight to 15 years [63]. The reductions in reported mercury levels have been explained by broadly applied domestic and international controls on chemicals and metals combined with dietary advice, although there is no scientific evidence for such effects. Linking global anthropogenic mercury emissions to mercury concentrations in the Arctic biota, and further how this affects the exposure to human populations who consume wildlife food, can be claimed to be one of the most important issues to be addressed when it comes to human health impacts in the Arctic. Table 2 presents an overview of the mercury concentrations in samples from Arctic populations.

**Figure 1:**
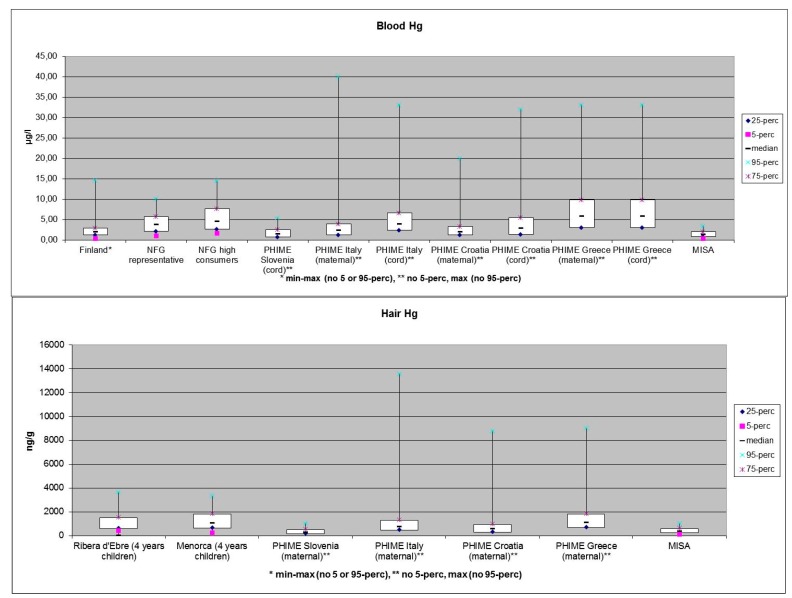
Overview of total mercury in blood (**Upper panel**) and hair (**Lower panel**) in the ArcRisk study groups [71].

The health research on mercury in the ArcRisk project focused on compiling and examining available information on mercury exposure and health effects in population groups in the Arctic and in the Mediterranean region. Data for the Mediterranean were used for comparison sake. In addition, data were used from the FP6 project PHIME [64]. The data of total mercury levels was comprised of hair or/and blood samples collected from several populations in Spain (Ribera d’Ebre, Menorca), Slovenia, Croatia, Greece, Italy, Norway (the Northern Norway mother-and-child contaminant cohort study—MISA and the Norwegian Fish and Game study—NFG), and northern Finland. Figure 1 presents a summary of the results of total mercury levels measured in the ArcRisk cohorts [65].

It is worth noticing that the ArcRisk studies (investigated in the PHIME cohorts) found that exposure to mercury did not have any negative effect on neurodevelopment at the age of 18 months. Exposure to higher mercury levels during pregnancy did not cause lower performances in cognitive, language and motor neurodevelopmental testing, since other correlating variables (e.g., socioeconomic indicators, maternal IQ) predict these outcomes. Higher fish consumption during pregnancy was associated with higher cognitive and language (but not motor) neurodevelopmental performance at 18 months of age thus emphasizing the benefits of including fish in the diet. Nevertheless, the ArcRisk project concluded that further studies are needed to evaluate the potential threat to European populations taking into consideration exposure to various mercury compounds and mixtures of stressors with similar end-points.

**Table 2:** Overview of mercury concentration in samples from Arctic populations.

Tissue	Species	Location	Year	Unit	Mean	Min	Max	N	Chemical Form	Source
**Spleen**	Inuit	*Greenland*	1990–1994	**µg/g**	**0.10**	0.031	0.31	*35*	**THg**	[66]
					**0.02**	0.0030	0.058	*32*	**MeHg**	
**Brain**		*Greenland*	-		**0.17**	0.059	4.8	*17*	**THg**	[67]
**Liver**		*Greenland*	1990–1994		**0.54**	0.087	1.5	*71*	**THg**	[66]
					**0.10**	0.022	0.21	*34*	**MeHg**	
**Kidney**		*Greenland*	1990–1994		**1.4**	0.024	4.9	*37*	**THg**	
					**0.046**	0.0080	0.11	*33*	**MeHg**	
**Urine**	General population	*Norway*	2003	**µg/g creatinine**	**1.3**	0.10	5.1	*178*	**IHg**	[68]
**Blood**	Women	*Nunavut-Baffin region*, *Canada*	1997	**µg/L**	**6.7**	0.10	34	*30*	**THg**	[69]
					**6.0**	0.80	29	*30*	**Organic Hg**	
			2005–2007		**4.0**	0.52	28	*99*	**THg**	
					**2.4**	0.20	23	*99*	**Organic Hg**	
		*North Slope*, *Alaska*	1999–2003		**1.1**	-	-	*43*	**THg**	[63]
		*Nunavik*, *Canada*	2007		**4.0**	0.70	24	*42*		
		*Nunavik*, *Canada*	2001		**9.9**	1.6	33	*19*		
		*Nunavik*, *Canada*	1997		**11**	3.8	44	*53*		
		*Nunavik*, *Canada*	1996		**13**	4.2	29	*25*		
		*Nunavik, Canada*	1992		**12**	3.6	33	*11*		
		*Disko Bay*, *Greenland*	2006		**12**	3.5	33	*20*		
		*Nuuk*, *Greenland*	2005		**3.2**	0.70	20	*10*		
		*All Greenland*	1999–2006		**13**	0.50	160	*299*		
		*Kola Peninsula*, *Russia*	2001–2003		**0.89**	0.50	2.4	*7*		
		*Krasnoschelye*, *Russia*	2001–2003		**3.3**	1.0	8.2	*47*		
		*Lovosero*, *Russia*	2001–2003		**7.3**	1.3	29	*11*		
**Blood**	Men	*Krasnoschelye*, *Russia*	2001–2003	**µg/L**	**5.4**	3.0	12	*4*	**THg**	[63]
		*Lovosero*, *Russia*	2001–2003		**6.6**	0.50	22	*9*		
		*Sisimiut*, *Greenland*	2002–2003		**6.5**	1.4	23	*52*		
		*Qaanaag*, *Greenland*	2003		**54**	2.3	240	*43*		
		*Nuuk*, *Greenland*	2005		**16**	3.0	52	*16*		
		*Quegertarsuaq*, *Greenland*	2006		**22**	3.5	79	*35*		
		*Narsaq*, *Greenland*	2006		**10**	3.0	25	*29*		
		*All Greenland*	1999–2006		**20**	1.4	240	*314*		
		*Norway*	2003		**16**	0.60	30	*184*		[68]
**Hair**	Children	*Qaanaaq*, *Greenland*	1995	**µg/g**	**5.5**	-	18	*43*	**THg**	[70]
	Women				**16**	-	33	*31*		
	3 year-old children	*Nunavut*, *Canada*	2007–2008		**1.6**	-	-	*142*		[71]
	Inuit children F		2007–2008		**1.3**	-	-	*161*		
	Inuit children M		2007–2008		**1.5**	-	-	*196*		
	Inuit children	*Kivallig region*, *Canada*	2007–2008		**1.1**	-	-	*133*		
		*Baffin region*, *Canada*	2007–2008		**2.1**	-	-	*158*		
		*Kitikmeot region*, *Canada*	2007–2008		**0.52**	-	-	*70*		

**THg**: Total mercury compounds; **MeHg**: methylmercury; **IHg**: inorganic mercury compounds; **N**: total number of samples.

## 3. What will be the Future Impacts of Energy and Climate Changes on the Contamination of the Arctic Ecosystems?

### 3.1. Energy and Technology Use Changes

Energy and technology use changes and their relevance for mercury emissions depend on many factors, including those connected to national and international initiatives to reduce greenhouse gases (GHGs). Improvement of efficiency of energy production in power stations, replacement of fossil fuels by renewable sources, and improvement of efficiency of emission control devices are likely to have a positive impact on the reduction of releases of mercury [2,9,72]. The International Energy Agency (IEA) has in the World Energy Outlook (WEO) and Energy Technology Perspectives (ETP) respectively, presented changes in energy production and consumption until the year 2035. In the projections, the WEO places focus on certain key aspects, such as energy prices, concerns for greenhouse gas emissions and its impacts on energy investments, the increasing use of renewable energy, changes in regulations and directives as well as recent developments in technologies for energy production. IEA further projects that the population for all the scenarios will increase from 6.7 billion (in 2008) to 8.5 billion (in 2035), population in non- OECD countries growing the most rapidly. In this period, the GDP on average is estimated to increase worldwide by 3.2% per year. India, China and Middle East are assumed to grow most rapidly in terms of GDP (Gross domestic Product) as well as increase in energy demand.

New mercury emission estimates (for year 2035) involving both activity and technology variables projected for the Current Policy (CP) scenario, New Policy (NP) scenario, and the Maximum Feasible Reduction (MFR) scenario were recently presented in the EU GMOS project [19]. The scenarios were based on assumptions from the IEA reports as well as previous results from the UNEP/AMAP, 2013 work. The CP Scenario assumed that governmental policies and measures existing in 2010 were adopted, including those that have not been fully implemented. The New Policies Scenario assumed that policy commitments and plans announced by countries worldwide to reduce greenhouse gas emissions, as well as phasing out fossil-energy subsidies, were going to be fully implemented. National climate commitments are related to the period of 2020, but additional measures were assumed to be implemented in the 2010 to 2020 pace for the period 2020 to 2035. The Maximum Feasible Reduction Scenario did set out a target of all counties reaching the highest feasible/available reduction efficiency in each emission sector. The assumptions for the energy sector were consistent with a 50% chance of limiting the average global temperature to 2 degree C (compared to pre- industrial levels). This requires the concentrations of greenhouse gases in the atmosphere of about 450 ppm of carbon dioxide equivalents. The scenario was not judged to be a very realistic one, but it illustrated the maximum possible mercury emission reductions that could be achieved if no other constraints are taken into account, such as economy and increased industry demand. The comparison of the EU GMOS future emission estimates indicated that if mercury continues to be emitted under the control measures and practices that are decided on today against a background of changing population- and economic growth, the 2010 emissions will remain the same in 2035 as in 2010. A full implementation of policy commitments and plans (the basic assumption of the NP scenario), implies a benefit of reducing global mercury emissions by almost 50% in 2035 under the assumptions employed in this scenario. A maximum feasible reduction of mercury emissions resulted in 85% less emissions than those envisaged under the CP scenario.

Irrespective of the above mentioned projections, Rafaj *et al.*, [73], provided projections for the year 2050 on the implications of renewable energy policies on atmospheric emissions of mercury in Europe using the GAINS model. They further found that current emissions of mercury in Europe are likely to remain at the same level under baseline conditions, but applying current standard air pollution control devices would mean a 35% reduction of mercury emissions. Low carbon policies and renewable electricity deployment would also in this study bring considerable co-benefit removal of mercury.

Changes in energy sector are expected to affect the mercury contamination in the Arctic. A relatively modest reduction of deposition is expected for the Arctic from emission control equipment as these installations effectively control emissions of particulate and reactive forms that tend to deposit close to the emission source. The Arctic reduction is expected to occur from long- range transport of gaseous elemental mercury. The major effect of the emission reductions under the scenarios is likely to occur in South- East Asia. Previous atmospheric modelling results have indicated that the annual flux of mercury to the Arctic will decrease by around 10% under assumptions comparable to the NP scenario mentioned above [5]. Descriptions of the 2020 scenarios are available in Pacyna *et al.* [72]. No further extrapolation of the results of atmospheric deposition decreases until 2020 was made for concentrations of mercury in various environmental media.

Even though the future climate change mitigation options are expected to reduce emissions and thus the long-range transport of gaseous elemental mercury, it is not clear how important these emission reductions are to the changes in mercury in the Arctic, compared to factors impacted by climate change.

### 3.2. Climate Change

#### 3.2.1. Effects on Environmental Fate and Behaviour

Previous research studies have indicated that climate change now and in the future will influence the environmental fate of mercury [1,4]. Climate change typically affects several physical factors such as those related to long- range transport from wind direction, precipitation rates, ocean currents, melting of polar ice caps and mountain glaciers, higher frequency of extreme events, and biotic transport. Moreover, enhanced levels of mercury reaching the Arctic may lead to severe human health damage as the Arctic human population reliance on traditional diets cause them to consume fish and other seafood with high concentrations of toxic forms of mercury (mainly methylated mercury) [5].

The mercury cycle in the Arctic is vulnerable to climate variability and change through its biochemistry and changes to the cryosphere. Increased Arctic air temperature lead to the acceleration in the loss of sea-ice cover, which leads to a warmer upper ocean and consequently to the change in the atmospheric connections between the Arctic and lower latitudes. Atmospheric mercury chemistry, including AMDEs (atmospheric mercury depletion events) may also be affected by changing temperatures and precipitation [5]. Temperature effects on the dependence on gaseous elemental mercury and bromine (Br) reactions or generation of bromine are also expected to affect AMDEs. Even though no studies are performed on low temperature dependence, theoretical calculations predict that the net oxidation of gaseous elemental mercury to HgBr2 will be much faster at cold temperatures, such as those observed in the Arctic [74]. It is thus expected that increased temperatures in the Arctic would slow down the mercury deposition if the concentrations of bromine are assumed to be the same.

Using the Danish Eulerian Hemispheric Model (DEHM) system, Christensen *et al.*, [75] modelled in a long temporal perspective the atmospheric deposition in the Arctic due to climate change by using climate meteorological data from the ECHAM5/MPI-OM (SRES A1b scenario) for year 1900–1999, 2090–2099 and 2190–2199 (with an assumption of constant 2000 emissions). They modelled 20% to 40% higher mercury deposition over continental areas in the Arctic and near Arctic under climate change scenarios, compared to present-day climate. 20% to 40% lower deposition rates were modelled directly to the Arctic Ocean by 2090–2099 compared to the 1990–1999. The changes in mercury deposition were expected to be driven by changes in the atmospheric chemistry of mercury that result from reduced sea-ice cover and increased concentrations of ozone in the troposphere that are forecasted under the climate change scenario [75].

For terrestrial systems increased temperature trends have led to a shorter period of ice and snow cover, reduced glaciers and permafrost, changed vegetation, river discharges, incidents of forest fire, and changes in migration pathways. A shorter period of ice and snow cover means that inorganic mercury through greater productivity are transformed to methylmercury in wetlands, marine and freshwaters [5]. Mercury methylation may increase with higher temperatures and thus higher bacterial activity in sediments. On the contrary can microbial processes lead to demethylation from higher temperatures, but in general, it is expected that the net methylation rate increase with higher temperatures. Since global warming is likely to extend the ice-free season, the season for mercury methylation is also increased.

Stern *et al.*, [4] concluded that the most important impacts of climate warming in the Arctic have occurred in precipitation rates, and type of precipitation (*i.e.*, rain or snow), river discharge and seasonality, lake ice and sea ice seasonality, thickness and extent, declining length and depth of snow cover, increasing active layer depth in permafrost soils, altered vegetation and drainage basins, and changing atmospheric connectivity between the Arctic and southern latitudes.

#### 3.2.2. Effects on Bioaccumulation

Through bioaccumulation mercury reaches higher concentrations in biota than are present in the environmental media in the first place. Under climate change, the structure and dynamics of marine food webs are in change. Arctic marine ecosystems are further affected by the reduction of sea- ice coverage and thickness [3]. The ability to adapt to ecosystem shifts depends on the individual species, so the effect on mercury bioaccumulation depends on the food-webs structures and their responses to climate change. Trophic structures vary geographically and occur in two ways; either from the “bottom-up”, or from the “top-down”. “Bottom-up” changes links to primary or secondary productivity, such as plankton and other organisms, stratification, nutrient supply, light intensity or ice cover [5]. These can have major effects on the production of organisms on a higher trophic level, but the complexity of such systems does not bring any confidence in future predictions under climate change. Changes in the top of the food- web, relate e.g., to the loss of sea ice cover and subsequent a shift in diet, habitats and/or migration pathways.

#### 3.2.3. Nutritional Transition and Change of Food Supply

Changed biotope conditions and thus changed availability of current marine fish and mammals used for human consumption, as well as increased exploitation of natural resources, may have an influence on human exposure to mercury. Climate change may affect fish supply and the associations between habitual diet and blood concentrations for many contaminants. Furthermore, it is a serious concern that climate change will cause changes that affect the supply of traditional foods such as marine mammals and reindeer for people living in the Arctic. It has been concluded that the traditional foods and local products are more nutritionally adequate than the imported foods replacing them. Severely limiting the consumption of fish and seafood may do more harm than good by reducing the consumption of foods with health benefits and by increasing the consumption of alternative foods that have potential health risks such as increasing the possibility of obesity and diabetes.

## 4. Final Discussion

### 4.1. Environmental Fate and Behaviour

Assessing the impact of future changes of climate on human health in the Arctic depends on the quality of information on energy projections and related mercury emission scenarios, environmental transport and inter-compartmental migration of mercury leading to environmental and human exposure in the future. The quality and accuracy of this information differ from adequate data for energy projects to very uncertain information on future human exposure. There is no linear relationships between the future changes of mercury emissions and changes of mercury in fish and seafood. This fact has been proven in previous AMAP assessments of mercury fate and behaviour in the Arctic. Similar conclusion has been reached in research carried out in the Great Lakes region in North America [76]. The mercury emissions in the Great Lakes region were reduced to one 10th between the beginning of the 1970’s and the beginning of the 2000’s. In the same period, there was only a two-fold reduction of mercury content in Herring Gull eggs on Snake Island, Lake Ontario [77]. The mercury emissions in this region are believed to originate almost entirely from local sources, and the fish is the main diet for birds in this region. One could therefore assume that even substantial reduction of mercury emissions may result in rather low reductions of mercury in fish. While chemical recovery of the environment due to emissions reductions could be measured as a short-term process, the biological recovery of the environment will take much longer time. This is particularly true in the case when climate change will result in volatilization of mercury already accumulated in soil and water (re-emission). This process is expected to emit more mercury to the atmosphere in the future compared with current re-emissions due to estimated air temperature rise. This enhanced re-emission may compensate for reduction of emissions from anthropogenic sources. Therefore, it could be stated that reductions of mercury emission from anthropogenic sources worldwide would need to be introduced as soon as possible in order to assure lowering the adverse impact of climate change on human health. The forthcoming new UNEP global emission reduction convention is expected to contribute to this reduction, although there are indications that implementation of various climate change mitigation options aimed at reducing greenhouse gas emissions, such as improvement of energy efficiency in power plants, replacement of fossil fuels by renewable sources, improvement of industrial technologies and abatement equipment, have a substantial positive effect on the reduction of mercury emissions [9,19,73].

### 4.2. Bioaccumulation

The bioaccumulation process for mercury in biota depends on structures in the food webs and their responses to climate change. It can be concluded that there are problems with our ability to estimate food-web bioaccumulation in Arctic marine food webs with state-of-the-art modelling tools under existing climate conditions. Predictions on how climate change will affect bioaccumulation is limited by the lack of understanding of the effects of climate change on methylation processes, primary production, species’ distribution and trophic interactions.

Regional considerations are moreover important for making meaningful predictions or evaluation of climate change impacts on mercury exposure in the Arctic. In northern Canada, patterns of environmental change are different in the eastern *vs*. western Arctic, with greater temperature change occurring in the west [74]. Further, temporal trends of mercury bioaccumulation in monitored animal populations vary regionally within the circumpolar Arctic, with a clear west-to-east gradient in the occurrence of recent increasing mercury trends [78]. These trends may be driven by different mercury sources from long-range transport and/or climate change. At the same time, there are indications that the signal in fish does not relate to the emissions, but rather to environmental factors (climate variables). Mercury methylation may be a key focus of change, prohibitive of the nutria transition and food supply may have a larger influence, meaning that whether regional shifts in global mercury emissions is reflected in Arctic biota mercury concentrations is currently unknown.

### 4.3. Nutritional Transition and Change of Food Supply

It is important to acknowledge that methylmercury exposure not only depends on contamination levels, but also on dietary habits and fish- and seafood species availability. The future risk of exposure will therefore in a large degree depend on nutritional transition and food supply. The amount of methylmercury ingested is becoming more uniform worldwide since there will be more global trade in fish and seafood. Although it is difficult to assess how the mercury intake through fish and seafood consumption will change under climate changing conditions, the fish stock in the Arctic may be affected, which is the main source of food in the region and a major pathway for mercury to enter the human body. Will a possible shortage of fish in the Arctic be compensated by other types of food or fish brought to the markets in the Arctic from other regions of the globe? Will this food be more contaminated than fish consumed in the Arctic today? It is difficult to address these questions with our current state of knowledge on climate change impacts on fish in the Arctic. Furthermore, it is not clear if such changes will be larger or smaller than climate related effects.

## 5. Conclusions

- To assess how climate change may alter the mercury exposure to Arctic populations is a very ambiguous task given the science currently available on the interactions between climate change and mercury cycling in the Arctic. Research indicate that climate change may act in both ways regarding decreasing and increasing effects. As discussed above, there is no linearity in relationships between the future changes of mercury emissions and changes of mercury in fish and seafood. It should be noted that although this paper has reviewed existing information, much more research is needed to further analyse the issue of climate change impact on the effects of mercury on human health in the Arctic.
- New methods would need to be developed or improved for the assessment of emissions reduction impacts, including models integrating mercury transport within the abiotic and biotic parts of the environment, mercury methylation and de-methylation process, dose-response functions for mercury in the populations and cost benefit analysis for selecting the most efficient emission reduction measures. Such knowledge should be further analysed in the context of improving current regulations and possibly definition of new legislation.
- Reductions of mercury emission from anthropogenic sources worldwide would need to be introduced as soon as possible in order to assure lowering the adverse impact of climate change on human health.
